# Long non-coding RNA CERS6-AS1 plays a prognostic role in promoting the progression of gastric cancer

**DOI:** 10.1080/21655979.2021.2012620

**Published:** 2021-12-22

**Authors:** Zhengliang Li, Xiaojing Liu, Nan Luo, Yali Pang, Yubin Hou, Guoxiang Jiang

**Affiliations:** a Second Department of Radiotherapy, Yantaishan Hospital, Yantai, China; b Department of Digestive Internal Medicine, Seventh People’s Hospital of Shanghai University of Tcm, Shanghai, China; c Third Department of Oncology, Weifang Hospital of Traditional Chinese Medicine, Weifang, China; d Catheter Room, Affiliated Hospital of Weifang Medical University, Weifang, China; e First Department of Surgery, Yantai Tao Cun Central Hospital, Yantai, China

**Keywords:** Gastric cancer, prognosis, lncRNA CERS6-AS1, miR-567

## Abstract

This study aims to investigate the potential clinical function of long non-coding RNA CERS6-AS1 (lncRNA CERS6-AS1) integrated miR-567 in gastric cancer. The expression of CERS6-AS1 in gastric cancer tissues was detected through RT-qPCR in contrast to the normal tissues. The correlation between the expression of lncRNA CERS6-AS1 and the characteristics of clinical data was analyzed. Kaplan-Meier curve was used to assess the survival analysis, while Cox proportional hazards model multivariate analysis was performed to evaluate the prognostic risk factors of gastric cancer to verify the prognostic possibility of CERS6-AS1. The expression of CERS6-AS1 in different gastric cancer cells was detected, being the development of gastric cancer cells after knockdown CERS6-AS1 studied using CCK-8, Transwell migration, and invasion detection methods. The targeting effect and interaction between CERS6-AS1 and miR-567 through biological analysis and luciferase activity detection. The expression of lncRNA CERS6-AS1 was elevated in gastric cancer tissues and cells. The results of this study demonstrate that the condition of gastric cancer patients was related to the expression of CERS6-AS1, and therefore CERS6-AS1 might be a prognostic factor for the progression of gastric cancer. In addition, the ability of gastric cancer cells to proliferate, migrate and invade could be reduced by knockdown CERS6-AS1. After CERS6-AS1 knockdown, the expression level of miR-567 in gastric cancer tissues decreased, while the expression level of miR-567 increased. In conclusion, lncRNA CERS6-AS1 might promote the progression of gastric cancer and had the potential as a prognostic marker of gastric cancer.

## Introduction

Gastric cancer (GC) is one of the most frequent cancers [1–3]. According to a recent study of 2020, gastric cancer has become the fifth most common cancer in the world [4]. Gastric cancer is characterized by a poor prognosis, high mortality, strong recurrence, and complex causes [5,6]. It has been reported several risk factors associated with gastric cancer, including bad eating and an unhealthy lifestyles, family genes, and living environment [7]. Based on current research, surgery is the best option for the treatment of this cancer [8,9]. However, most patients are diagnosed at the late stage of gastric cancer, and the recurrence rate of surgery is high, and the patients’ condition is unstable [10].

Long non-coding RNAs (lncRNAs), a class of RNA that lack protein-coding capacity and more than 200 nucleotides long, have been shown to play an important role in cell growth and differentiation, and many cancers [11,12]. MicroRNAs (miRNAs) are a type of RNA molecule with a length of about 20 nucleotides, which cannot be used for protein coding [13]. LncRNAs can specifically target and bind miRNAs to regulate the progression of cancer [14,15]. In this context, new evidence suggests that lncRNA may affect the progression of gastric cancer. The development of lncRNA-guided prognostic methods for gastric cancer may be established since the overexpression of lncRNA HOTAIR may be used as a biomarker for a poor prognosis of gastric cancer [16]. Zhang et al confirmed that lncRNA MT1JP, as a down-regulated lncRNA, may regulate the progression of gastric cancer, suggesting that MT1JP may become a potential therapeutic target and a prognostic marker for gastric cancer [17]. In addition, through exploring the molecular mechanism, it has been found that lncRNA CERS6-AS1 may accelerate the proliferation and growth of cells and slow down the progress of cell apoptosis in breast cancer [18]. However, no detailed studies exist on the mechanism of action of lncRNA CERS6-AS1 in gastric cancer.

The present study gradually verified the mechanism of action of CERS6-AS1 through the analysis and detection of the expression of lncRNA CERS6-AS1 and miR-567 in gastric cancer tissues and cells and the clinical relationship. On the other side, the possibility of lncRNA CERS6-AS1 targeting miR-567 on the growth and prognosis of gastric cancer cells was determined by knockdown CERS6-AS1 and the study of interaction between CERS6-AS1 and miR-567.

## Materials and methods

### Characteristics of the patients

Patients with gastric cancer (N = 126) were randomly selected and divided into high expression group (n = 66) and low expression group (n = 60) according to the mean expression of lncRNA CERS6-AS1. Table 1 listed the details of the clinical data of all the included patients. This research selected patients with gastric cancer diagnosed and treated by the Seventh People’s Hospital of Shanghai University of TCM from February 2014 to November 2016. All the included patients signed an informed consent form under the supervision of the ethics committee, ensuring that no treatments that would affect the results of the research were taken in advance.

**Table 1.** Correlation of the lncRNA CERS6-AS1expression with clinical characteristics in GC

Indicators	Cases (n = 126)	lncRNA CERS6-AS1 expression		*P*
		Low (n = 60)	High (n = 66)	
Age				0.598
≤ 55	64	29	35	
> 55	62	31	31	
Gender				0.165
Male	76	40	36	
Female	50	20	30	
Tumor size(cm)				0.054
<5	57	33	25	
≥5	68	27	41	
Local invasion				0.269
T1,T2	67	35	32	
T3,T4	59	25	34	
Differentiation				0.149
Well, Moderate	65	35	30	
Poor	61	25	36	
Lymph node metastasis				<0.001
Negative	66	49	17	
Positive	60	11	49	
TNM stage				<0.001
I, II	71	45	26	
III, IV	55	15	40	

Annotation: GC: gastric cancer.

### Cell culture and transfection assay

Gastric cancer cells BGC-823, AGS, HGC-27, and SGC-7901 and control cells GES-1 were purchased from the Institute of Biochemistry and Cell Biology, Chinese Academy of Sciences (Shanghai, China). All experimental cells were cultured in Dulbecco’s Modified Eagle’s medium (DMEM; Invitrogen, USA) including 10% fetal bovine serum (FBS; USA), which were incubated in a 37°C cell incubator (DRH-A100, Shanghai, China) under 5% CO_2_ conditions [19].

Cell transfection was performed using Lipofectamine 2000 (Invitrogen, Carlsbad, USA). In the beginning, gastric cancer cells were finely inoculated in 6-well plates for static culture. After 48 hours, the HGC-27 and AGS gastric cancer cells were successfully transfected to construct the knockdown test group si-CERS6-AS1 and the control group si-NC.

### Reverse transcription-quantitative polymerase chain reaction (RT-qPCR)

TRIzol (Invitrogen, USA) method was selected to obtain total RNA. DNase I reagent (Takara, Japan) was adopted to further purify the extracted RNA. Primescript Reverse Transcriptase (Takara, Japan) was used to obtain cDNA that can be RT-qPCR. SYBR Premix (Takara, Japan) was used to configure the reaction system for RT-qPCR. Glyceraldehyde 3-phosphate dehydrogenase (GAPDH) and U6 were defined as internal references. The data collected was effectively processed and analyzed by the 2^−ΔΔCt^ method. The primers involved were as follows: CERS6-AS1 forward 5ʹ-GCAGCCCAGCAGAAGTAG

GA-3ʹ and reverse 5ʹ-GAGCATAGGGAAGCAACTCTCAG-3ʹ; GAPDH forward 5ʹ-CATCACTGCCA

CCCAG-3ʹ and reverse 5ʹ-ATGCCAGTGAGCTTCCC-3ʹ; U6 forward 5ʹ-CTCGCTTCGGCAGCACA-3ʹ and reverse, 5ʹ-AACGCTTCACGAATTGCGT-3ʹ.

### Cell counting Kit-8 and Transwell assay

The transfected cells were seeded in a 96-well plate with a seeding density of 2 × 10^3^ cells/mL, and continued to be cultured in a 37°C incubator. At the specified time point, 10 μL of Cell Counting Kit-8 (CCK-8) reagent (Dojindo Laboratories, Japan) was placed on the plates [20]. After mixing the reagent and the cells thoroughly, the absorbance of each well was detected at the wavelength of 450 nm, and the average value taken.

The cell migration operation was to add DMEM medium to the upper part of Transwell (Corning, China) and DMEM/F12 medium to the lower part [21]. After 48 hours of co-cultivation, the cells were washed twice with phosphate buffered saline (PBS), and then fixed with glutaraldehyde at 4°C. The cells were stained with crystal violet for 30 min before the observation in the microscope. The operation of the invasion assay is the same as the above-mentioned method, but Matrigel (Becton Dickinson, USA) was added to the upper part of the Transwell before the assay.

### Luciferase activity detection

The pmirGLO vector (Promega, Shanghai, China) was used to construct WT-CERS6 - AS1 (wild-type) and MUT-CERS6-AS1 (mutated-type). Lipofectamine 2000 (Invitrogen, USA) was used to co-transfect HGC-27 cells with WT-CERS6-AS1 or MUT-CERS6-AS1 and miR-567 mimic, mimic NC, miR-567 inhibitor, inhibitor NC, or control. After 48 hours, the dual-luciferase reporter gene detection system (Program, USA) was selected to measure the luciferase activity.

### Statistical analysis

SPSS 20.0 version (IBM, USA) was performed for data collation and analysis, being the results were expressed as mean ± standard deviation (SD). The χ^2^ test was used to show the relevant clinical data of patients with gastric cancer. The Kaplan-Meier curve was used to assess the survival analysis. Cox proportional hazards model multivariate analysis was used to evaluate the prognostic risk factors of gastric cancer, aiming to study the potential use of CERS6-AS1 as a prognostic biomarker for gastric cancer.

## Results

The expression level of CERS6-AS1 in gastric cancer tissues and cells was detected by RT-qPCR, aiming to explore the potential of CERS6-AS1 as a prognostic biomarker for gastric cancer. Kaplan-Meier curve and Cox analysis were conducted to evaluate the prognostic value of CERS6-AS1. The functional assays, including CCK-8 assay and Transwell assays, were chosen to investigate the functional role of CERS6-AS1 in gastric cancer, and the dual-luciferase reporter gene assay confirmed the function of gastric cancer and the targeting effect of CERS6-AS1 on miR-567.

### Expression of lncRNA CERS6-AS1 in gastric cancer tissue

**Figure 1.**
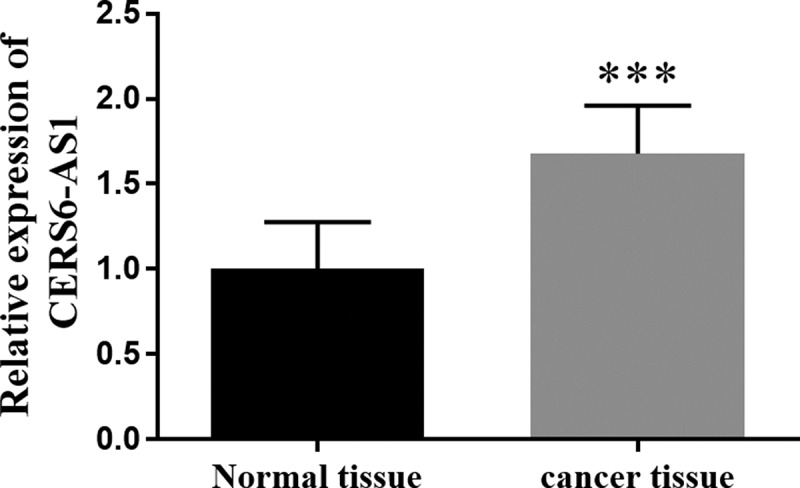
The relative expression of lncRNA CERS6-AS1 in gastric cancer tissues was increased compared with normal tissues by RT-qPCR. ***P < 0.001

The RT-qPCR method is often chosen to detect gene expression levels. The expression of CERS6-AS1 in gastric cancer tissues and normal tissues is shown in Figure 1. From Figure 1 it can be visualized that the expression of CERS6-AS1 in gastric cancer tissues was markedly higher in comparison with normal tissues, with an increase of about 70%.

Through the analysis of the clinical characteristics of 126 gastric cancer patients, it can be concluded that the expression of CERS6-AS1 is not only related to lymph node metastasis, but also has a significant correlation with tumor-node-metastasis (TNM) stage (*P* < 0.001). C20 Dec 2021ontrastingly, patient age, gender, gastric cancer tumor size, local invasion, and differentiation did not have a huge impact on the expression of CERS6-AS1(Table 1).

### Survival analysis of gastric cancer

**Figure 2.**
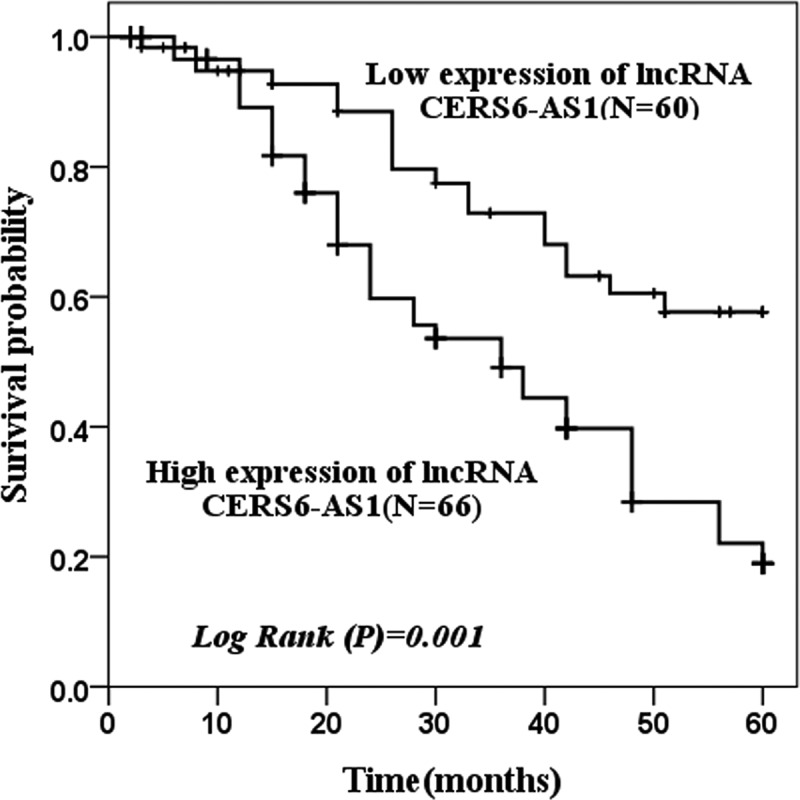
The survival probability of 60 months of low expression of lncRNA CERS6-AS1 and high expression of lncRNA CERS6-AS1 was analyzed by the Kaplan-Meier method. The survival condition of low expression of lncRNA CERS6-AS1 was significantly higher than that of a high expression of lncRNA CERS6-AS1 (log-rank *P* = 0.001)

Kaplan-Meier curve was used to assess the survival status of patients. Figure 2 shows that the survival probability with low expression of CERS6-AS1 (n = 60) and high expression of CERS6-AS1 (n = 66) in 60 months was analyzed. The group with a low expression of CERS6-AS1 had better survival and greater survival probability, while the group with a high expression of CERS6-AS1 had the opposite results (log rank *P* = 0.001).

The prognostic factors of gastric cancer were determined by multivariate Cox analysis of clinical features and overall survival, and the prognostic possibility of CERS6-AS1 was verified. As shown in Table 2, lncRNA CERS6-AS1 (*P* < 0.001), lymph node metastasis (*P* = 0.026) and TNM stage (*P* = 0.020) are all factors that affect the survival status of gastric cancer patients. Moreover, Cox analysis proved that lncRNA CERS6-AS1 may be used as a pr20 Dec 2021 20 Dec 2021 ognostic biomarker for gastric cancer.

**Table 2.** Multivariate Cox analysis of clinical characteristics in relation to overall survival

Indicators	Multivariate analysis		
	HR	95% CI	*P*
LncRNA CERS6-AS1	4.828	2.416–9.648	<0.001
Age	1.056	0.605–1.843	0.847
Gender	1.200	0.688–2.093	0.521
Tumor size	1.545	0.901–2.651	0.114
Local invasion	1.252	0.718–2.181	0.428
Differentiation	1.269	0.729–2.208	0.399
Lymph node metastasis	2.104	1.094–4.044	0.026
TNM stage	2.053	1.119–3.768	0.020

### Research assay of CERS6-AS1 in gastric cancer cells

**Figure 3.**
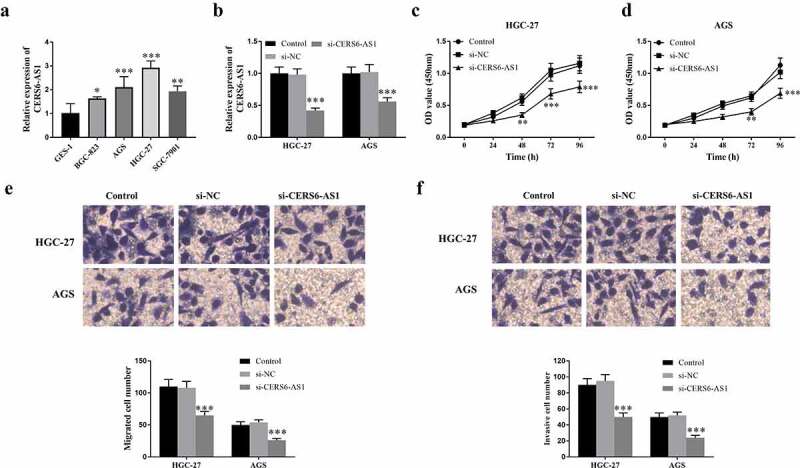
Expression level of lncRNA CERS6-AS1 in different gastric cancer cell lines and analysis of transfection, proliferation, migration and invasion in gastric cancer cells HGC-27 and AGS. (a) The relative expression level of CERS6-AS1 is upregulated in different gastric cancer cells, compared with normal cells GES-1. (b) The relative expression level of CERS6-AS1 in HGC-27, and AGS cells transfected with si-CERS6-AS1 was significantly downregulated. (c) and (d) Proliferative capacity of HGC-27 and AGS cells were reduced that measured by CCK-8. (e) The Transwell assay showed that the migration ability of HGC-27, and AGS cells was down-regulated. (f) The Transwell assay showed that the invasion level of HGC-27, and AGS cells was reduced. ***P < 0.001

The expression of CERS6-AS1 was detected in selected gastric cancer cells BGC-823, AGS, HGC-27, SGC-7901, and normal cells GES-1. According to Figure 3(a), the results proved that the expression of CERS6-AS1 determined by RT-qPCR was up-regulated in four gastric cancer cells. The gastric cancer cells HGC-27 and AGS with the relatively highest expression of CERS6-AS1 were selected for follow-up experiments. Figure 3(b) shows that knockdown CERS6-AS1 (si-CERS6-AS1) markedly decreased compared with si-NC and the control group. The results of the research on the proliferation ability of HGC-27 and AGS cells are shown in Figures 3c **and** 3d. Detecting the optical density (OD) value of knockdown CERS6-AS1 at the wavelength of 450 nm by CCK-8 shows that the proliferation ability of gastric cancer cells was down-regulated. Figure 3(e) shows the measurement results of migration ability it can be seen that the number of si-CERS6-AS1 cells was lower than that of the control groups, indicating that the migration ability of cancer cells will decrease with the knockdown of CERS6-AS1. In the same way, the Transwell method was used to detect the invasion ability of gastric cancer cells, and the results were in agreement, as shown in Figure 3(f).

### Determination of luciferase activity and the interaction between CERS6-AS1 and miR-567

**Figure 4.**
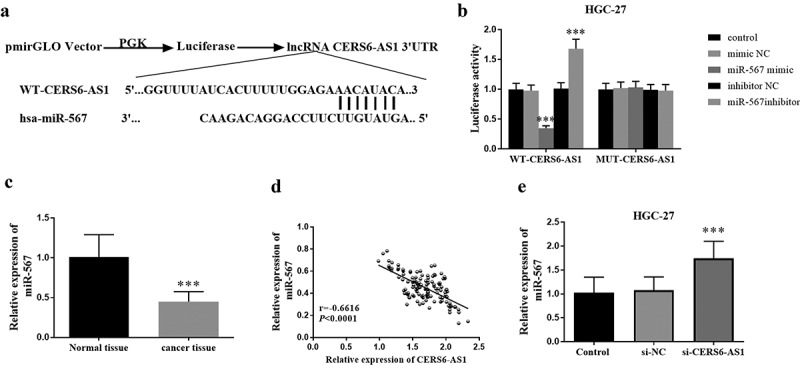
Luciferase reporter and the effect of CERS6-AS1 on miR-567. (a) The binding site of WT-CERS6-AS1 and miR-567. (b) Luciferase activity of WT-CERS6-AS1 and MUT-CERS6-AS1 in HGC-27 cells. (c) The miR-567 in gastric cancer tissues was down expressed compared with normal tissues by RT-qPCR. (d) The relative expression levels of CERS6-AS1 and miR-567 are negatively correlated. (e) The si-CERS6-AS1 obtained by knockdown CERS6-AS1 in HGC-27 cells increased the content of miR-567. ***P < 0.001

In this study, pmirGLO was selected as the vector to construct WT-CERS6-AS1 and MUT-CERS6-AS1. The binding sites of WT-CERS6-AS1 and miR-567 are shown in Figure 4(a), which were obtained by bioinformatics analysis. Through the detection of luciferase activity, it can be found from Figure 4(b) that in wild-type WT-CERS6-AS1, the expression of miR-567 (miR-567 mimic) increases the luciferase activity, while the expression of miR-567 (miR-567 inhibitor) inhibits the increase of luciferase activity. The difference is explained since the mutant MUT-CERS6-AS1 is not affected by the expression level of miR-567 in HGC-27 cells. The expression of miR-567 was detected in gastric cancer tissues. As shown in Figure 4(c), the expression of miR-567 in cancer tissues decreased in comparison with normal tissues. Further study on the interaction between CERS6-AS1 and miR-567, Figure 4(d) shows that CERS6-AS1 and miR-567 are negatively correlated. Thus, as the content of CERS6-AS1 increases, miR-567 gradually decreases. Figure 4(e) shows that in gastric cancer cell HGC-27, the relative expression of miR-567 in si-CERS6-AS1 was significantly increased. Therefore, the knockdown of CERS6-AS1 should promote the expression of miR-567.

**Figure 5.**
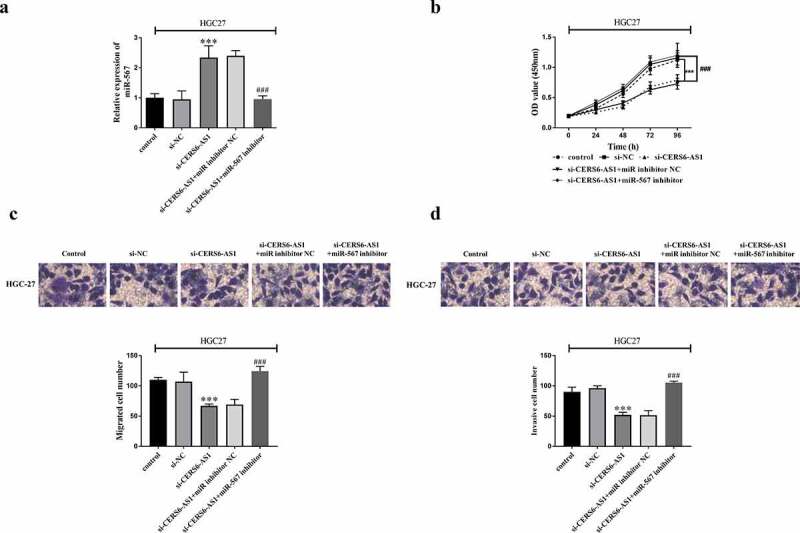
Co-regulated HGC-27 cells via the interaction between si-CERS6-AS1 and miR-567. HGC-27 cells were co-transfected with control, si-NC, si-CERS6-AS1, si-CERS6-AS1+ miR inhibitor NC or si-CERS6-AS1+ miR-567 inhibitor. (a) Expression of miR-567 was measured using RT-qPCR in HGC-27 cells. (b) Cell proliferation was measured by CCK-8. (c) Cell migration was analyzed using Transwell. (d) Cell invasion was analyzed using Transwell. ***P < 0.001 vs si-NC, ^###^*P* < 0.001 vs si-CERS6-AS1+ miR inhibitor NC

The relationship between CERS6-AS1 and miR-567 was further verified by knockdown of CERS6-AS1 recovery assay. HGC-27 cells were co-transfected with control, si-NC, si-CERS6-AS1, si-CERS6-AS1+ miR inhibitor NC or si-CERS6-AS1+ miR-567 inhibitor via Lipofectamine 2000. RT-qPCR results in Figure 5(a) shows that i-CERS6-AS1+ miR-567 inhibitor reversed the promotion of miR-567 expression by knockdown CERS6-AS1. The proliferation of HGC-27 cells was detected by CCK-8 method, and the results are shown in Figure 5(b). Cell proliferation level was down-regulated by knockdown CERS6-AS1, but the down-regulated expression was offset after co-transfection of miR-567 inhibitor, and the cells proliferated significantly. Figures 5c **and** 5d clarifiy that decreased migration and invasion ability of HGC-27 cells was reversed after the participation of si-CERS6-AS1+ miR-567 inhibitor, and cell viability was enhanced.

## Discussion

Gastric cancer is a malignant tumor that originates from the epithelium of the gastric mucosa, and its incidence varied according to the regions [22,23]. Studies have shown that the prevalence in males is higher than in females [24–26]. In our country, the early gastric cancer symptoms are not obvious, and the symptoms often show nonspecific features, resulting in a low diagnosis rate. With the development and progress of society, people’s irregular eating and life behaviors contribute to the earlier gastric cancer appearance [27]. Therefore, paying attention to the early diagnosis, treatment, and prognosis of gastric cancer is of great significance to the development of human health.

In the current study, RT-qPCR was used to detect the expression of CERS6-AS1, being found that the content of CERS6-AS1 in gastric cancer tissues increased and showed high expression, and the same was true in gastric cancer cells. In the study of breast cancer by Yan et al, it was found that CERS6-AS1 may improve the proliferation of breast cancer through miR-125a-5p, and the expression of CERS6-AS1 is up-regulated in breast cancer tissues [28]. Subsequently, CERS6-AS1 was also confirmed to show high expression levels in different pancreatic cancer cell lines [25]. According to the clinical characteristics of gastric cancer patients and Cox analysis, it can be inferred that lncRNA CERS6-AS1 may be a prognostic factor of gastric cancer. Studies have shown that lncRNA can be used for gastric cancer research. For example, lncRNA MAGI2-AS3 was previously proven to be an independent prognostic factor for the survival of gastric cancer patients [29]. The latest research also stated that lncRNA HCP5 may be a new and promising target for the treatment of gastric cancer [30]. Additionally, this study conducted a Kaplan-Meier curve analysis on the survival status of gastric cancer patients. After a 60-month follow-up survey, it can be seen that when CERS6-AS1 expression is low and the probability of survival is higher. Similarly, in the study of Bao et al the Kaplan-Meier survival curve also described the relationship between CERS6-AS1 expression and survival time in breast cancer patients [18]. Yun et al also studied the correlation between the expression of CERS6-AS1 and the overall survival of patients with pancreatic ductal adenocarcinoma [31].

It has been reported that knockdown of LINC00265 could inhibit the proliferation of gastric cancer cells in vitro [32]. Another study found that the high expression of LINC00511 could promote the proliferation and migration of gastric cancer cells [33]. After evaluating the related expression of CERS6-AS1 in gastric cancer tissues in this study, the effects of CERS6-AS1 deletion on cell proliferation, migration, and invasion were identified. Evidence shows that deletion of CERS6-AS1 can also inhibit the proliferation ability, migration level, and invasion ability of gastric cancer cells. Thus, when CERS6-AS1 is low expressed, the progression of gastric cancer is inhibited and the survival possibility is improved, which is consistent with the results of previous studies on the survival status of patients. Experiments have also shown that knockdown LIFR-AS1 inhibited gastric cancer cell proliferation capacity, invasion level, and migration ability, and induced gastric cancer cell apoptosis [34]. Finally, through biological analysis, such as luciferase activity detection, it was demonstrated that miR-567 was negatively adjusted by lncRNA CERS6-AS1. When the level of CERS6-AS1 decreased, the expression of miR-567 was up-regulated. Similarly, the study of Xu et al about the mechanism of CERS6-AS1 on pancreatic cancer concluded that CERS6-AS1 also negatively adjusted miR-217 [25]. The literature shows that lncRNA CERS6-AS1 is used in the research and analysis of a variety of cancer processes, such as hepatocellular carcinoma [35], pancreatic cancer [25,36], breast cancer [28], and most of them are related to the poor prognosis of tumors by targeting miRNAs. In other words, CERS6-AS1 has been verified herein to play a role in the gene regulation of miR-567, and might be used as a potential biomarker for prognosis assessment.

## Conclusion

In conclusion, the results demonstrated in this study clarified that the lncRNA CERS6-AS1 knockdown inhibited the proliferation and growth of cancer cells and other biological functions, while CERS6-AS1 negatively regulates miR-567 to affect the progress of cancer. Thus, lncRNA CERS6-AS1 has the potential to play a prognostic role in promoting the progression of gastric cancer.

## Data Availability

The corresponding author can provide relevant data for this study.

## Highlights

(1) The tissues and cells CERS6-AS1 in gastric cancer was upregulated.

(2) CERS6-AS1 might be a promising prognostic biomarker of gastric cancer.

(3) Knockdown of CERS6-AS1 could repress the progression of gastric cancer through miR-567 regulation.
